# Knockdown of PDX1 enhances the osteogenic differentiation of ADSCs partly via activation of the PI3K/Akt signaling pathway

**DOI:** 10.1186/s13018-021-02825-4

**Published:** 2022-02-19

**Authors:** Fan Liu, Guang-Dong Chen, Long-Kun Fan

**Affiliations:** 1 The First Department of Anesthesiology, Cangzhou Central Hospital, Cangzhou, 061000 China; 2 The Department of Orthopedics, Cangzhou Central Hospital, No. 16 Xinhua West Road, Cangzhou, 061000 China; 3 The Department of Medical Plastic Surgery, Cangzhou Central Hospital, Cangzhou, 061000 China

**Keywords:** PDX1, Adipose derived stem cells, Osteogenic differentiation, Bioinformatic analysis

## Abstract

**Background:**

Osteoporosis (OP) is a systemic bone disease manifested as low bone mass, destruction of bone microstructure, increased bone fragility and fracture risk. The purpose of this study was to explore the role and mechanism of PDX1 for osteogenic differentiation of adipose derived stem cells (ADSCs).

**Methods:**

GSE37329 dataset was retrieved from NCBI Gene Expression Omnibus (GEO) database and performed bioinformatic analyses. ADSCs were incubated with normal medium, osteogenic induction medium (OIM) and OIM+si-PDX1. Then, alkaline phosphatase (ALP) staining and Alizarin Red Staining (ARS) were performed to assess the role of PDX1 for osteogenesis of ADSCs. PI3K inhibitor, LY294002 was then added to further explore the mechanism of PDX1 for osteogenic differentiation of ADSCs. Western blot assay was used to assess the osteogenic-related markers. Graphpad software was used to perform statistically analysis.

**Results:**

A total of 285 DEGs were obtained from analysis of the dataset GSE37329, of which 145 were upregulated and 140 were downregulated genes. These differentially expressed genes mainly enriched in cell differentiation and PI3K/Akt signaling pathway. Moreover, PDX1 was decreased in osteogenic induced ADSCs. Knockdown of PDX1 significantly increased osteogenic differentiation capacity and p-PI3K and p-Akt protein levels. Administration with LY294002 could partially reversed the promotion effects of si-PDX1.

**Conclusion:**

In conclusion, knockdown of PDX1 promotes osteogenic differentiation of ADSCs through the PI3K/Akt signaling pathway.

**Supplementary Information:**

The online version contains supplementary material available at 10.1186/s13018-021-02825-4.

## Background

Osteoporosis is a condition in which bone becomes weak which is characterized as low bone mass and structural deterioration [1]. As a result, bone tissue becomes fragile and shows increased vulnerability to fracture [2]. Worldwide, osteoporosis affects about 200 million people and is often unrecognized until one encounters the fracture due to the silent nature of the disease [3]. Under healthy conditions, bone is maintained by the constant process of bone remodeling. Normal bone remodeling maintains a balance between bone resorption and formation to maintain bone density [4]. Osteoblasts are generated from the osteogenic differentiation of mesenchymal stem cells (MSCs) [5]. MSCs possess the capacity to self-renew and to differentiate into multiple cell types. It has been known that MSCs are common precursors for osteoblasts [6]. The direction of MSC differentiation depends on specific regulatory factors.

Many factors influenced bone formation by affecting osteogenic differentiation through targeting several pathways in ADSCs. Previously, we found that miR-1249-5p regulates the osteogenic differentiation of ADSCs by targeting PDX1 [7]. Role and mechanism of the PDX1 in regulating osteogenic differentiation of ADSCs remains unknown. Pancreatic and duodenal homeobox factor 1 (PDX1) is the most important transcription factor in cell differentiation of multiple stem cells [8, 9]. However, the role of PDX1 in regulating osteogenic differentiation of ADSCs was unknown.

Many pathways were associated with osteogenic differentiation of ADSCs, including Wnt/β-catenin [10], PI3K/Akt [11, 12] and p38-MAPK [13] signaling pathways. PI3K/Akt signaling pathway was one of the most important pathway that involved into cell differentiation [14]. Using a bioinformatics approach, we analyzed the signaling pathway through PDX1 target genes and constructed a protein–protein interaction network.

In this study, we firstly identified the differentially expressed genes between osteogenic induced ADSCs and non-induced ADSCs through GSE37329 datasets. Moreover, following experiments were performed to identify the mechanism of PDX1 in regulating osteogenic differentiation of ADSCs.

## Materials and methods

### Bioinformatic analysis

GSE37329 was downloaded from Gene Expression Omnibus including 3 ADSCs, 2 ADSCs-derived osteocytes. Background correction and normalization were performed with package 'affy' of R. Missing values were filled with the median method. Differentially expressed genes were identified using the DESeq2 R package and subsequent analysis of gene expression was performed in R. Both heatmap and volcano plots were constructed to present the expression profiles of differentially expressed genes using hierarchical clustering, which was performed using the R software. Gene Ontology (GO) and Kyoto Encyclopedia of Genes and Genomes (KEGG) pathway enrichment analysis was performed using the R package clusterprofiler for genes, to identify over-represented GO terms in three categories (biological processes, molecular function and cellular component), and KEGG pathway. A protein–protein interaction network was acquired using the STRING database with the standard setting. Subnetwork models were selected using the plugin molecular complex detection (MCODE) application in Cytoscape 3.6.1 software (Cytoscape Consortium, California, USA).

### ADSCs isolation and culture

All experiments were confirmed by the Ethical Committees of the Cangzhou Central Hospital. ADSCs were isolated from human adipose tissue obtained from patients who were undergoing total knee arthroplasty at the Department of orthopedic, the XXX Hospital. Adherent cells were cultured in a growth medium [DMEM/F12 (HyClone, USA), 10% FBS, 1% Penicillin‐Streptomycin Solution (Gibco, USA)] at 37 °C/5% CO_2_ and saturated humidity. ADSCs were passaged after reaching 90% confluence. The antibodies including anti-CD45-FITC, anti-CD45-PE, anti-CD90-FITC and ant-CD105-PE were purchased from BD biosciences (USA). Flow cytometry was used to test these surface markers of mesenchymal stem cells at passage three.

Multi-lineage potential assay was performed to identify osteogenic, adipogenic and chondrogenic phenotype. In brief, ADSCs were cultured into 6-well plates and then transferred to adipogenic medium (Cyagen Biosciences co., LTD, Guangzhou, China), osteogenic medium (Cyagen Biosciences co., LTD, Guangzhou, China) or chondrogenic medium (Cyagen Biosciences co., LTD, Guangzhou, China). At the end of the culture time, ADSCs were stained with ARS, Oil-Red-O staining and Alcian blue staining. Photographs were taken in a light microscope.

### Osteogenic differentiation

The differentiation of cultured ADSCs was induced by incubating osteogenic medium containing BMP-2, FBS (5%), β-glycerophosphate (3 mM), and ascorbic acid (50 μg/mL) for 7, 14 days. For osteogenic differentiation assays, 2 × 10^4^ cells were seeded in triplicates in a 24-well format plate and cultured in complete medium. Cells maintained in normal growth media were used as the control.

### RT-PCR and real-time PCR

Cells were collected for gene expression profiling related to osteogenic differentiation. To perform qRT-PCR, RNA was isolated using Trizol (Invitrogen). cDNA was produced from 2 μg RNA with reverse transcriptase as described by the M-MLV manual (New England Biolabs). For RT-PCR, PDX1 primers were used. The relative quantity of PDX1 was normalized to GAPDH. The expression of PDX1 was detected using qPCR with SYBR Green Mix Kits (Applied Biosystems). All results were quantitated using the 2^−ΔΔCt^ relative quantification method.

### Western blot analysis

Total proteins were extracted with lysis buffer containing PMSF and RIPA (PMSF: RIPA = 1: 99) and then qualified with the Bicinchoninic Acid (BCA) assay kit (Beyotime, Beijing, China). Thereafter, protein extracts (20–40 μg) were separated on a 10% sodium dodecyl sulfate polyacrylamide gel electrophoresis, transferred onto a nitrocellulose membrane and then blocked with 5% milk for 1 h. Next, the membrane was probed with primary antibodies overnight at 4 °C, followed by the corresponding secondary antibody for 1 h at 37 °C. The blots were visualized using enhanced chemiluminescence reagents (BeyoECL Plus; Beyotime Institute of Biotechnology). All antibodies were bought from Abcam (Cambridge, MA, USA): OSX (1:5000), OCN (1:5000), ALP (1:5000), RUNX2 (1:2000), and GAPDH (1:5000).

### PDX1 knockdown

The siRNA target sequence was designed against human PDX1. For transient transfection, PDX1 sequence was retrieved from GenBank, and two siRNA sequences were designed, namely, PDX-1 siRNA-1 (5ʹ-AGCCAAGAGAGGCATAGA-3ʹ), PDX-1 siRNA-2 (5ʹ-AGCTACCCGGAAGACAG-3ʹ). Transfection efficiency was controlled by evaluating PDX1 level using RT-PCR and Western Blot analysis after 48 h.

For the knockdown experiment, 50% confluent ADSCs were transfected using Scrambled siRNA and designed PDX1-specific siRNAs using Lipofectamine RNAiMAX reagent (Invitrogen, USA). The efficiency of knockdown of PDX1 was confirmed 2 days after siRNA transfection. To further assess the PDX1 for the influence of PI3K/Akt signaling pathway, LY294002 at a concentration of 10 μM and 50 μM for 12 h before exposure to OIM according to previous literature [15]. The inhibitor efficacy was further identified by western blot to assess the protein level of p-Akt. PDX1 overexpression plasmid (Flag- PDX1) was purchased from Origene Technologies (Origene).

### Alkaline phosphatase (ALP) activity

After treatment, each plate was fixed by 4% paraformaldehyde for 10 min and then washed with PBS for three times. Cells were stained with 5-bromo-4-chloro-3-indolylphosphate (BCIP) and 4-nitro blue tetrazolium chloride (NBT) for 30 min. Then the reactions were stopped with distilled water. Observation and photographing were performed by a light microscope (Olympus, Milan, Italy). ALP activity was measured and the results were normalized to levels of total protein.

### Alizarin red S staining

Matrix mineralization was assessed through staining with Alizarin Red S (Solarbio, Beijing, China). After stimulation, the cells were washed, followed by fixation with ethanol (70%) for 45 min. Cells were then stained with the dye Alizarin Red S. Fluorescence signals were visualized using a fluorescence microscope.

### Statistical analysis

All experiments were performed in triplicate. The data were displayed as the mean ± standard deviation (SD) and processed by GraphPad Prism 7 software. Student’s t test or one-way analysis of variance (ANOVA) was utilized to analyze significant differences. The *P* < 0.05 indicated statistically significant.

## Results

### Bioinformatic analysis results

**Fig. 1:**
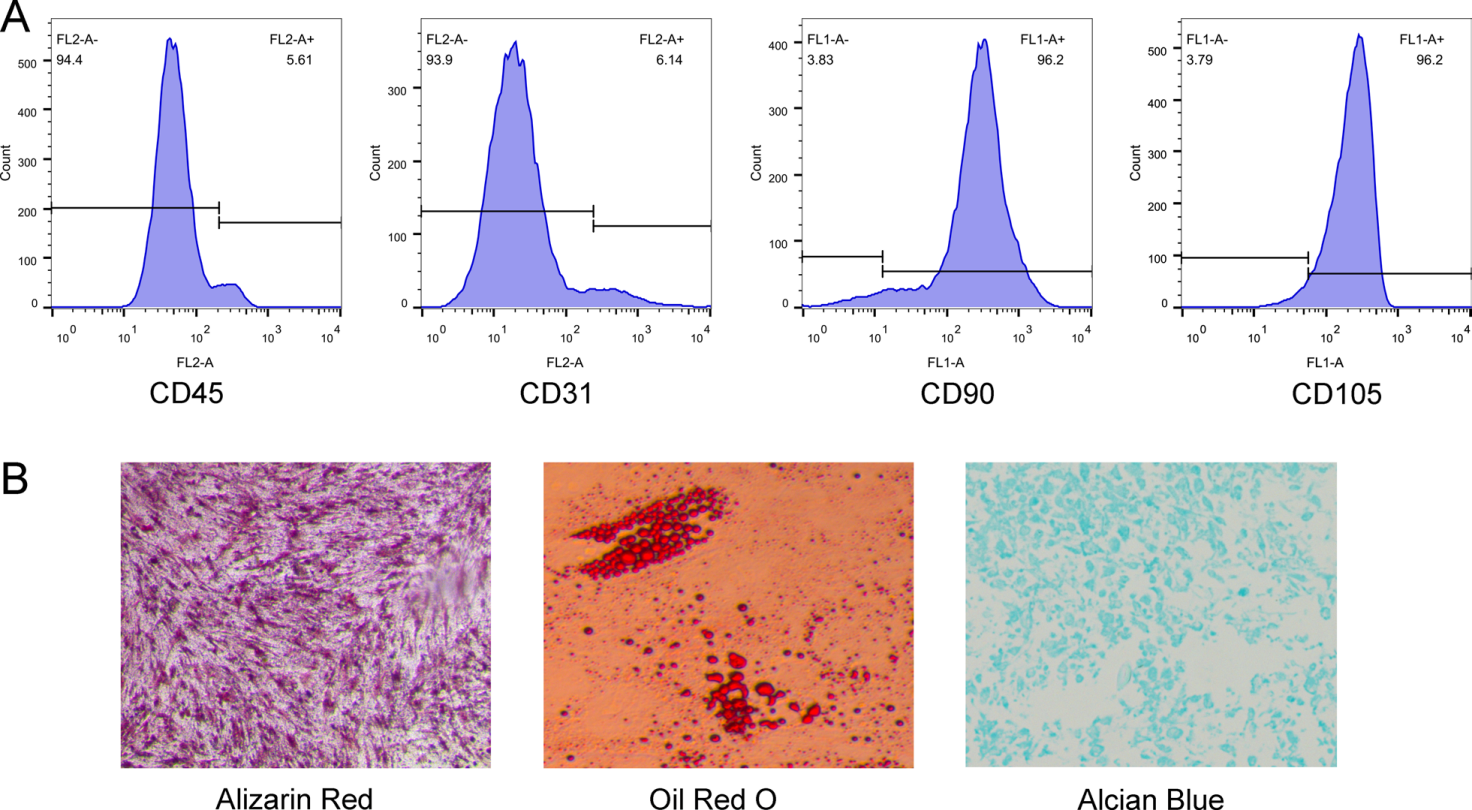
Identification of human ADSCs. **A** Flow cytometric analysis of ADSC surface markers (CD45, CD31, CD90 and CD105). **B** Osteogenic, adipogenic and chondrogenic differentiation of ADSCs

**Fig. 2:**
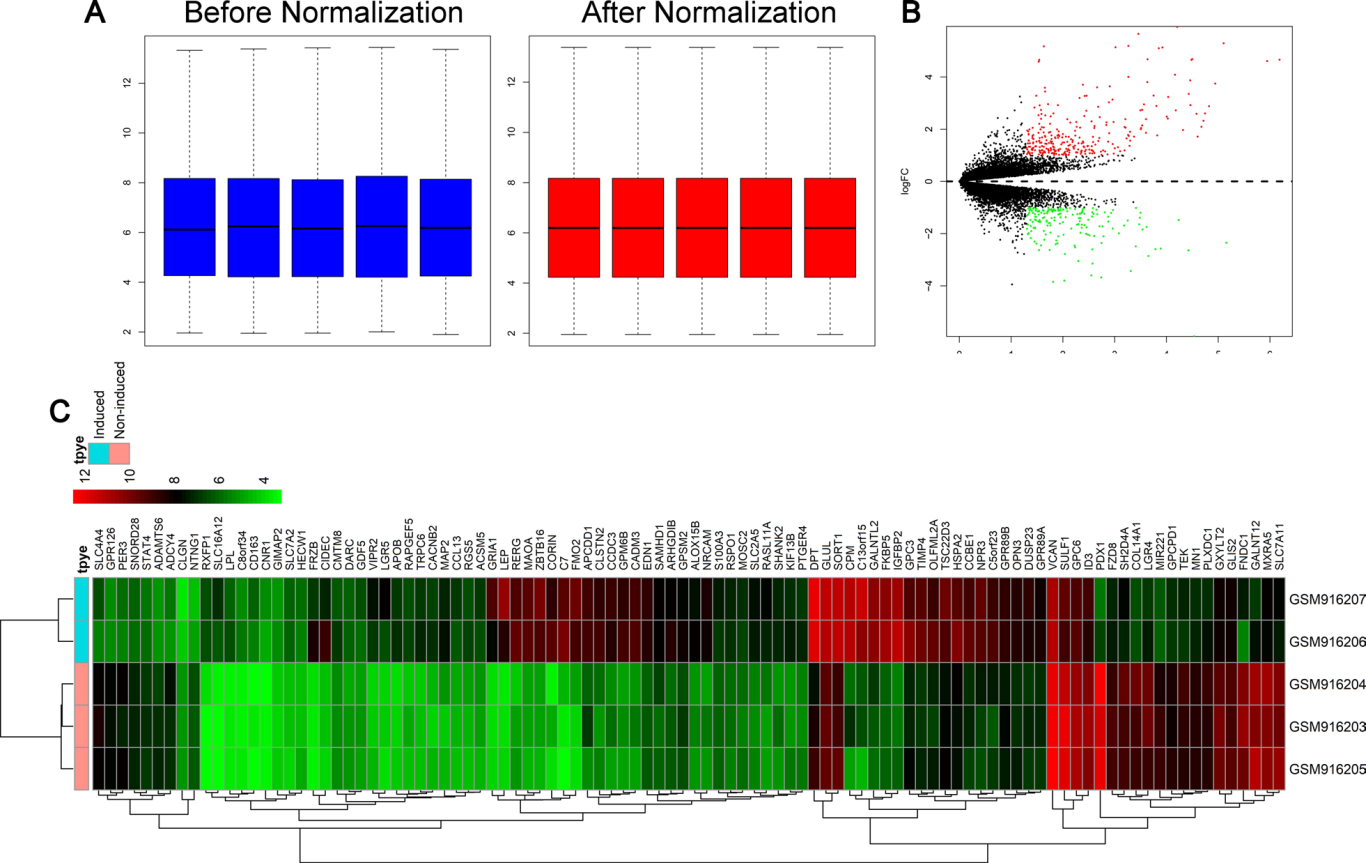
Bioinformatic analysis of GSE37329 dataset. **A** Comparison of expression value between before normalization and after normalization; **B** the volcano plot of the differentially expressed genes in GSE37329; Red dots represent upregulated genes, green dots represent downregulated genes and black dots represents non-differentially expressed genes. **C** Clustering heatmap of differentially expressed genes. Red color indicates up-regulated gene while green color indicates down-regulated gene

Box plots before and after normalization of the raw data is shown in Fig. 1A. The median values of each sample were extremely similar, indicating that the data should be further analyzed. A total of 285 DEGs were obtained from analysis of the dataset GSE37329, of which 145 were upregulated and 140 were downregulated genes. The volcano plot and heatmap of differentially expressed genes is presented in Fig. 2B and C respectively. we found the gene list to be enriched in biological processes including 204 genes mainly involved in response to lipopolysaccharide, extracellular matrix organization, cell–cell signaling, positive regulation of cytosolic calcium ion concentration, signal transduction, negative regulation of cell proliferation, negative regulation of transcription from RNA polymerase II promoter, fat cell differentiation, positive regulation of macrophage derived foam cell differentiation and negative regulation of smooth muscle cell proliferation.

Cellular component mainly enriched in extracellular space, extracellular region, proteinaceous extracellular matrix, plasma membrane, integral component of plasma membrane, extracellular exosome, cell surface, organelle membrane, platelet alpha granule lumen, anchored component of membrane. Molecular function terms mainly enriched in cytokine activity, heparin binding, chemorepellent activity, receptor binding, growth factor activity, transforming growth factor beta receptor binding, PDZ domain binding, Wnt-protein binding, transcription corepressor activity and N,N-dimethylaniline monooxygenase activity. KEGG pathways mainly enriched in the cGMP-PKG signaling pathway, neuroactive ligand-receptor interaction, signaling pathways regulating pluripotency of stem cells, PI3K/Akt signaling pathway, vascular smooth muscle contraction, TGF-beta signaling pathway, NF-kappa B signaling pathway, inflammatory mediator regulation of TRP channels, circadian entrainment and Rap1 signaling pathway.

Figure 1C represents the protein–protein interaction network of differentially expressed genes having 18 nodes and 86 edges. As illustrated in Fig. 1D, the top two functional clusters of modules were selected (module 1, MCODE score = 18.357; module 2, MCODE score = 10.333).

### Identification of ADSCs

**Fig. 3:**
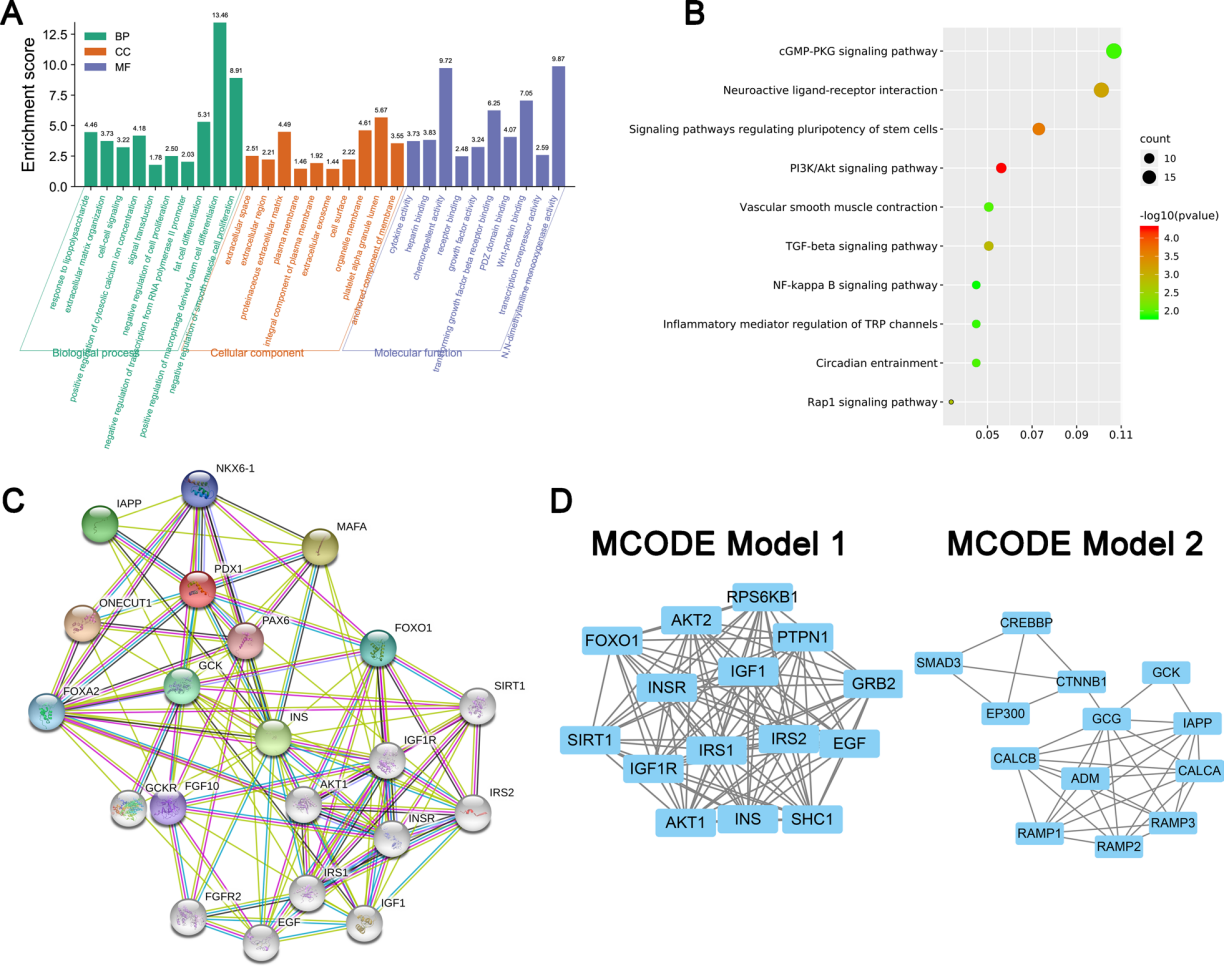
Gene function of the differentially expressed genes in GSE37329 dataset. **A** Gene ontology analysis of genes based on differentially expressed genes. Gene ontology was divided into biological process (BP, green color), cellular component (CC, orange color) and molecular function (MF, blue); **B** KEGG pathway analysis of the top 10 KEGG enriched gene pathway-related diseases. **C** Protein–protein interaction network; **D** top 2 MCODE model of the protein–protein interaction network

As illustrated in Fig. 2A, ADSCs expressed stem cell-associated markers CD90 and CD105 and negative expressed with CD45 and CD31. Under proper stimulation, ADSCs should be able to differentiate into osteogenic, adipogenic and chondrogenic progenies (Fig. 2B). Compared with control group, ADSCs that underwent osteogenic differentiation possess higher expression of PDX1 with statistically significant (*P* < 0.05, Fig. 3).

### Knockdown of PDX1 increase the osteoblastic differentiation capacity of ADSCs

**Fig. 4:**
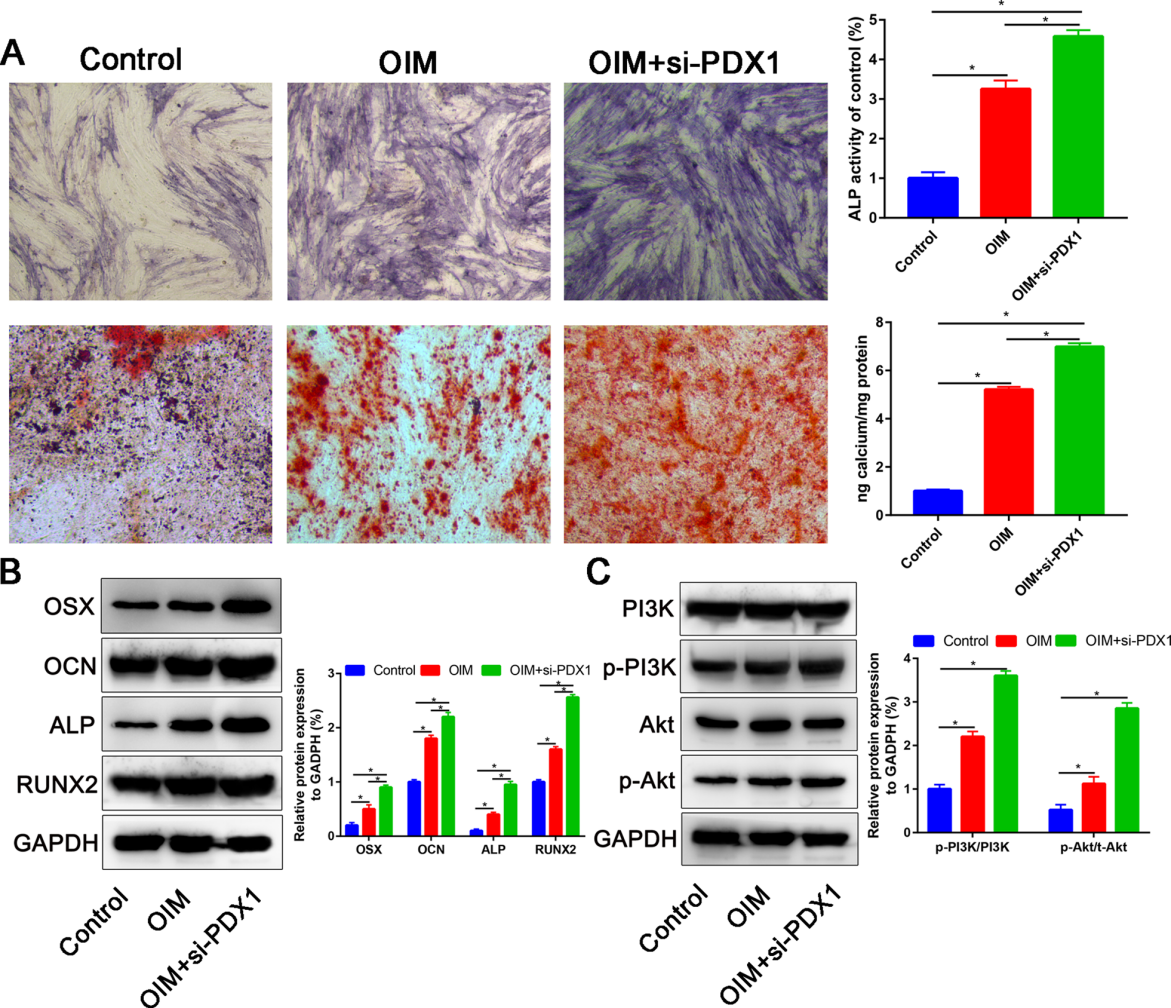
Knockdown of PDX1 could enhanced the osteogenic capacity of the ADSCs. **A** ALP activity and calcium deposition between control, OIM and OIM+si-PDX1. **B** Western blot assay that assess the OSX, OCN, ALP and RUNX2 in control, OIM and OIM+si-PDX1 groups. **C** PI3K, p-PI3K, Akt and p-Akt protein expression in in control, OIM and OIM+si-PDX1 groups. *P < 0.05

After transfection with PDX1 siRNA (PDX1 siRNA-1 or PDX1 siRNA-2), the PDX1 mRNA and protein expression was significantly downregulated (Additional file 1: Fig. S1A and B). ADSCs were divided into three groups: control, OIM and OIM+si-PDX1 groups. Then, ALP staining and ARS were performed to assess the early and late osteogenic ability respectively. Compared with control group, ADSCs cultured with OIM significantly increase the ALP activity and calcium deposition at 7 and 21 days, respectively. However, when administration with si-PDX1, the ALP activity and calcium deposition was partially attenuated than OIM alone group (Fig. 4A). Then, we performed western blot assay to assess the osteogenic-related markers (OSX, OCN, ALP and RUNX2) in each group. ADSCs cultured with OIM significantly increase the OSX, OCN, ALP and RUNX2 expression, while co-cultured with si-PDX1 partially attenuated these osteogenic-related markers expression (Fig. 4B). To further explore the mechanism of PDX1 for osteogenic differentiation of ADSCs, we assessed the PI3K/Akt pathway related markers change. We found that after knockdown of PDX1 could significantly activate the PI3K/Akt signaling pathway. ADSCs were transfected with PDX1 siRNA or control siRNA (control) for different times. Then, we assessed the p-PI3K and p-Akt expression in different treatment groups. In terms of increasing incubation duration, p-PI3K and p-Akt showed a progressive increasing trend (Additional file 2: Fig. S2).

### Overexpression PDX1 decrease the osteoblastic differentiation capacity of ADSCs

**Fig. 5:**
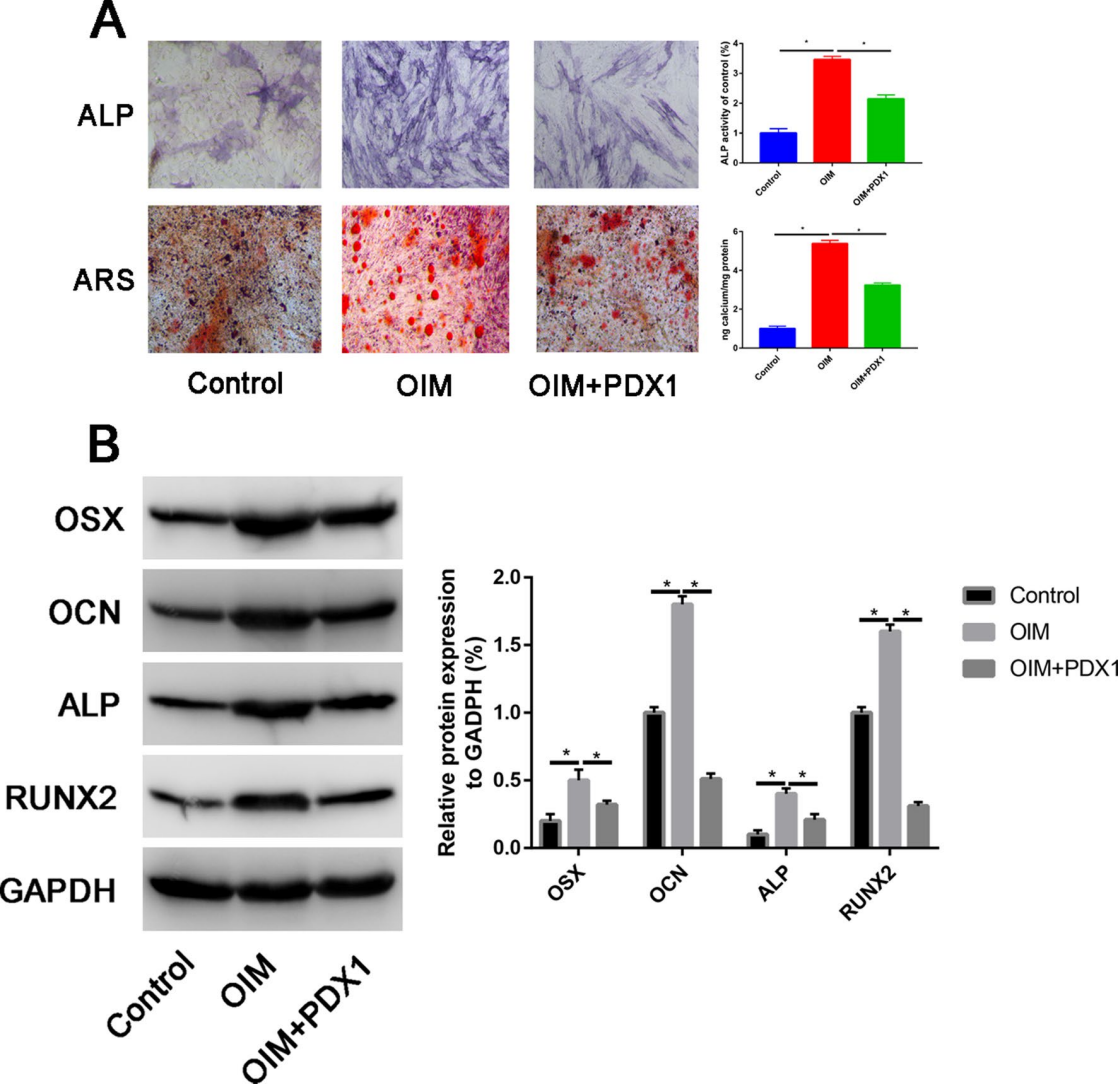
Overexpression PDX1 decrease osteogenic differentiation capacity of ADSCs. **A** ALP activity and calcium deposition in control, OIM and OIM+PDX1; **B** OSX, OCN, ALP and RUNX2 protein expression in control, OIM and OIM+PDX1. *P < 0.05

After transfection with PDX1 plasmid, ALP activity and calcium deposition was partially decreased (Fig. 5A, *P* < 0.05). Consistent with ALP activity and ARS staining, the OSX, OCN, ALP and RUNX2 were significantly downregulated in PDX1 group than OIM group (Fig. 5B, *P* < 0.05).

### LY294002 could partially reversed the promotion effects of si-PDX1

To further explore the mechanism of PDX1 for osteogenic differentiation of ADSCs. We administrated PI3K inhibitor, LY294002 to assess the osteogenic differentiation ability of ADSCs. Western blot showing that p-AKT expression decreased with LY294002 treatment (Additional file 3: Fig. S3). There was no significant difference between 10 μM and 50 μM LY294002 (*P* > 0.05).

**Fig. 6:**
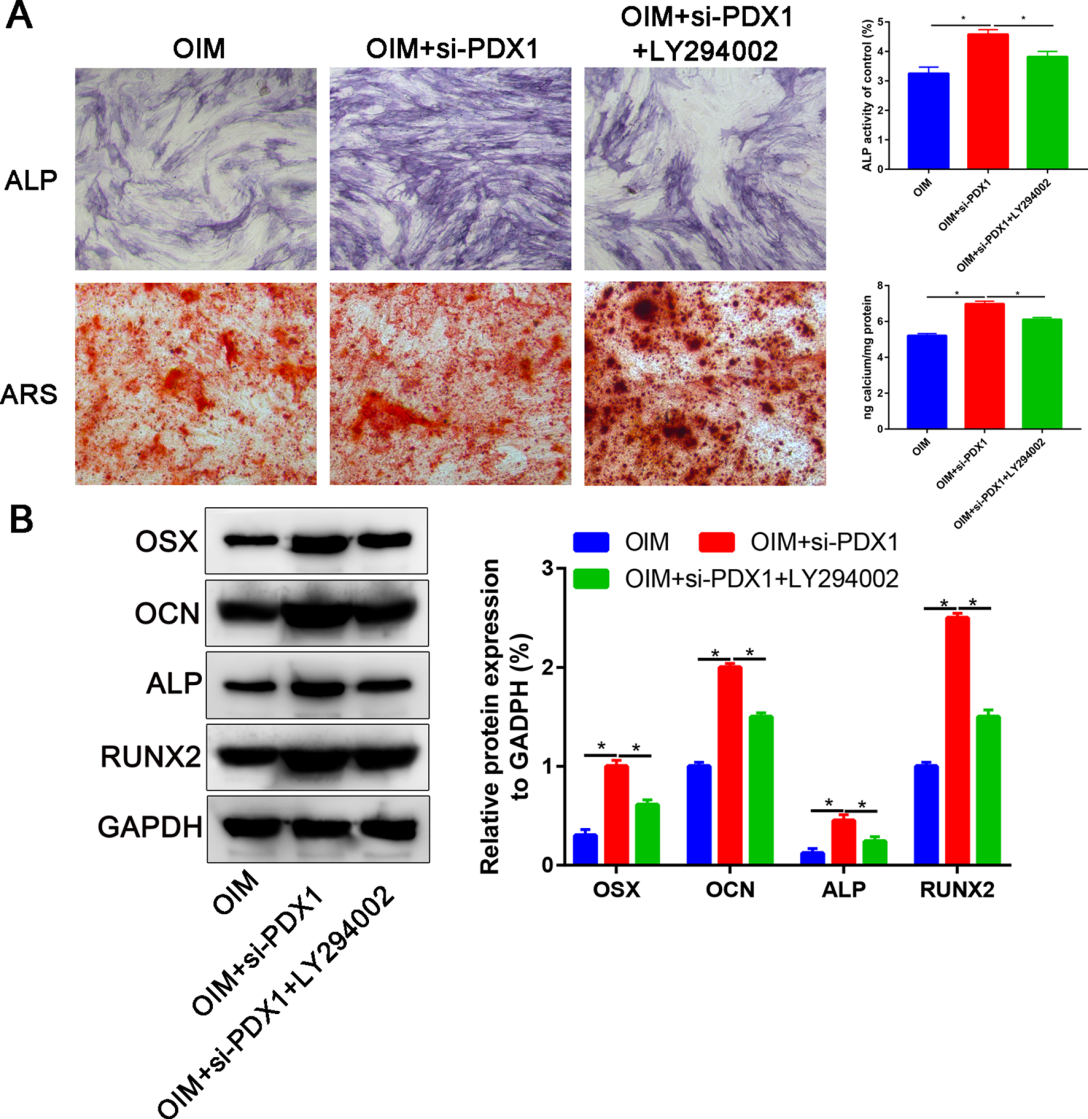
LY294002 partially block the promotion effects of si-PDX1 on osteogenic differentiation capacity. **A** ALP activity and calcium deposition in OIM, OIM+si-PDX1 and OIM+si-PDX1+LY294002; **B** OSX, OCN, ALP and RUNX2 protein expression in OIM, OIM+si-PDX1 and OIM+si-PDX1+LY294002. *P < 0.05

ALP and ARS staining found that administration LY294002 could partially reverse the ALP activity and calcium deposition after OIM induction (Fig. 6A). Further western blot assay found that LY294002 could partially downregulated the osteogenic related markers expression (OSX, OCN, ALP and RUNX2, Fig. 6B).

## Discussion

In this study, we firstly assessed the role of PDX1 for the osteogenic differentiation of ADSCs. We found that PDX1 was differentially expressed between OIM and normal medium. Further studies found that knockdown of PDX1 significantly increase the osteogenic capacity of ADSCs. Moreover, administrated with PI3K inhibitor could partially reversed the promotion effects of si-PDX1.

We firstly analyses the GSE37329 datasets, which containing 2 osteogenic induced ADSCs and 3 non-induced ADSCs. Further in-depth studies revealed that PDX1 may interact with the PI3K/Akt signaling pathway to promote the osteogenic differentiation of ADSCs. ADSCs are multipotent stem cells, which can differentiate into osteoblasts, odontoblasts and adipocytes in specific medium. ADSCs have become promising seed cells for bone tissue engineering due to their easy access and availability in large quantities [16]. It has been reported that ADSCs tend to differentiate into adipocytes rather than osteoblasts [17].

For ADSCs-based therapy to be successful in in vivo treatment of bone diseases, it is crucial to transplant ADSCs with a suitable substance that facilitates their osteogenic differentiation in vivo [18, 19]. Firstly, knockdown of PDX1 could significantly increase the osteogenic capacity, which suggested that PDX1 is emerging as a promising therapeutic target in bone disease therapy. PDX1 is a regulator of pancreas development and β cell differentiation [20]. Further study found that the increase in PDX1 levels is crucial for the development and differentiation of pancreatic β cells [21]. Moreover, PDX1 is a critical transcription regulators for beta cell development and regeneration [22].

Several signaling pathways are involved in ADSCs osteogenic differentiation, including the ERK1/2 [23], Wnt [6], phosphatidylinositide-3 kinase (PI3K)/Akt [24], and BMP-Smad pathways [25]. However, the mechanism of PDX1 for osteogenic differentiation of ADSCs is largely unknown. We found that PDX1 knockdown significantly activate the PI3K/Akt signaling pathway. Previously, Jara et al. [26] found that PDX1 contributes to β-cell mass expansion and proliferation induced by Akt/PKB pathway. These results suggested that PDX1 directly or indirectly affect the PI3K/Akt signaling pathway. In this study, we found that knockdown of PDX1 significantly activate the PI3K/Akt signaling pathway and finally promote the osteogenic differentiation of ADSCs. The PI3K/AKT pathway plays a crucial role in osteogenic differentiation of ADSCs and acts as a chief regulator in bone cells' proliferation and metabolism. Our data revealed that si-PDX1 through PI3K/AKT signaling pathways trigger osteogenic differentiation of ADSCs.

As revealed in the present study, the knockdown of PDX1 increased the levels of p-Akt and p-PI3K via the PI3K/AKT pathway in ADSCs, which was decreased by treatment with the PI3K inhibitor LY294002. Previous studies have revealed that enhanced osteoinductivity was induced through activating the PI3K/Akt signaling pathway [27–29]. Protein–protein interaction revealed that PDX1 could directly regulating PI3K/Akt signaling pathway. Western blot assay and PI3K inhibitor experiments further confirmed that PDX1 could affect the PI3K/Akt signaling pathway and finally influence the osteogenic differentiation of ADSCs.

The main limitation of the present study was the lack of in vivo experiments. Moreover, the downstream signaling pathways were not explained in detail. Multiple pathways involved into the osteogenic differentiation of ADSCs and the correlation between these pathways need to be identified.

## Conclusion

In conclusion, this study firstly demonstrated that knockdown of PDX1 could enhance the osteogenesis of ADSCs in vitro through PI3K/Akt signaling pathway. PDX1 was identified as novel target of bone-related disease. Our study provided a new insight into the treatment of OP.

## Supplementary Information

**Additional file 1**. A, Relative PDX1 expression in siRNA control, PDX-1 siRNA-1 and PDX-1 siRNA-2 groups.

**Additional file 2**. Relative p-PI3K, PI3K, p-Akt and Akt expression in control and PDX-1 treatment group with different treatment.

**Additional file 3**. Relative Akt and p-Akt expression in control and LY294002 (10μM and 50 μM) groups.

## Data Availability

We state that the data will not be shared since all the raw data are present in the figures included in the article.

## Abbreviations

OP: Osteoporosis
ADSCs: Adipose derived stem cells
GEO: Gene expression omnibus
OIM: Osteogenic induction medium
ALP: Alkaline phosphatase
ARS: Alizarin red staining
MSCs: Mesenchymal stem cells
PDX1: Pancreatic and duodenal homeobox factor 1
GO: Gene ontology
KEGG: Kyoto encyclopedia of genes and genomes
MCODE: Molecular complex detection
BCA: Bicinchoninic acid
BCIP: 5-Bromo-4-chloro-3-indolylphosphate
NBT: 4-Nitro blue tetrazolium chloride
SD: Standard deviation
ANOVA: One-way analysis of variance
