# Supplementary figures and images for: Knockdown of PDX1 enhances the osteogenic differentiation of ADSCs partly via activation of the PI3K/Akt signaling pathway
Source: J Orthop Surg Res. 2022 Feb 19;17:107. doi: 10.1186/s13018-021-02825-4 (PMC8858563; doi:10.1186/s13018-021-02825-4)

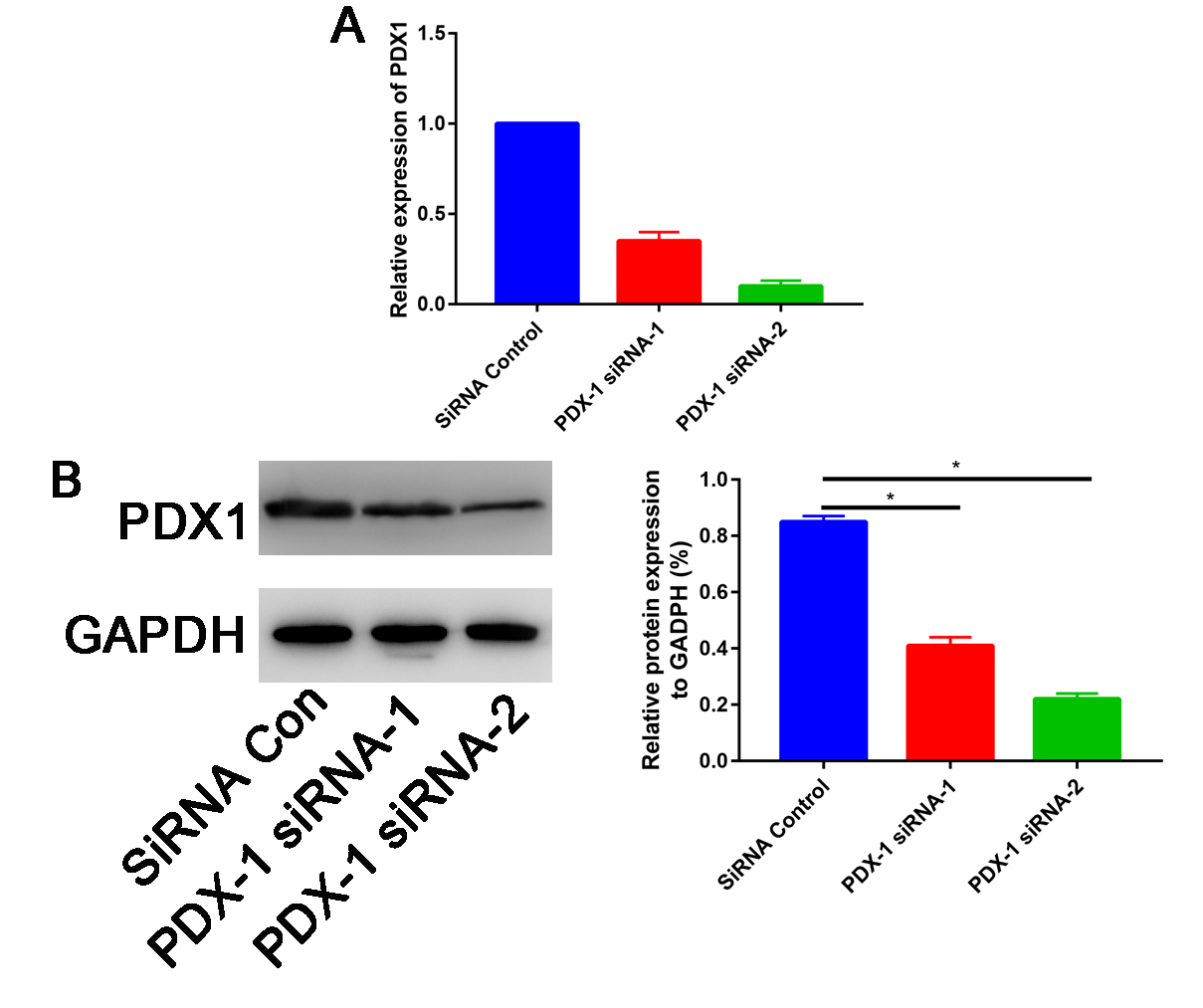

Supplement: Supplementary file 1 — Additional file 1. A, Relative PDX1 expression in siRNA control, PDX-1 siRNA-1 and PDX-1 siRNA-2 groups. [file 13018_2021_2825_MOESM1_ESM.tif]

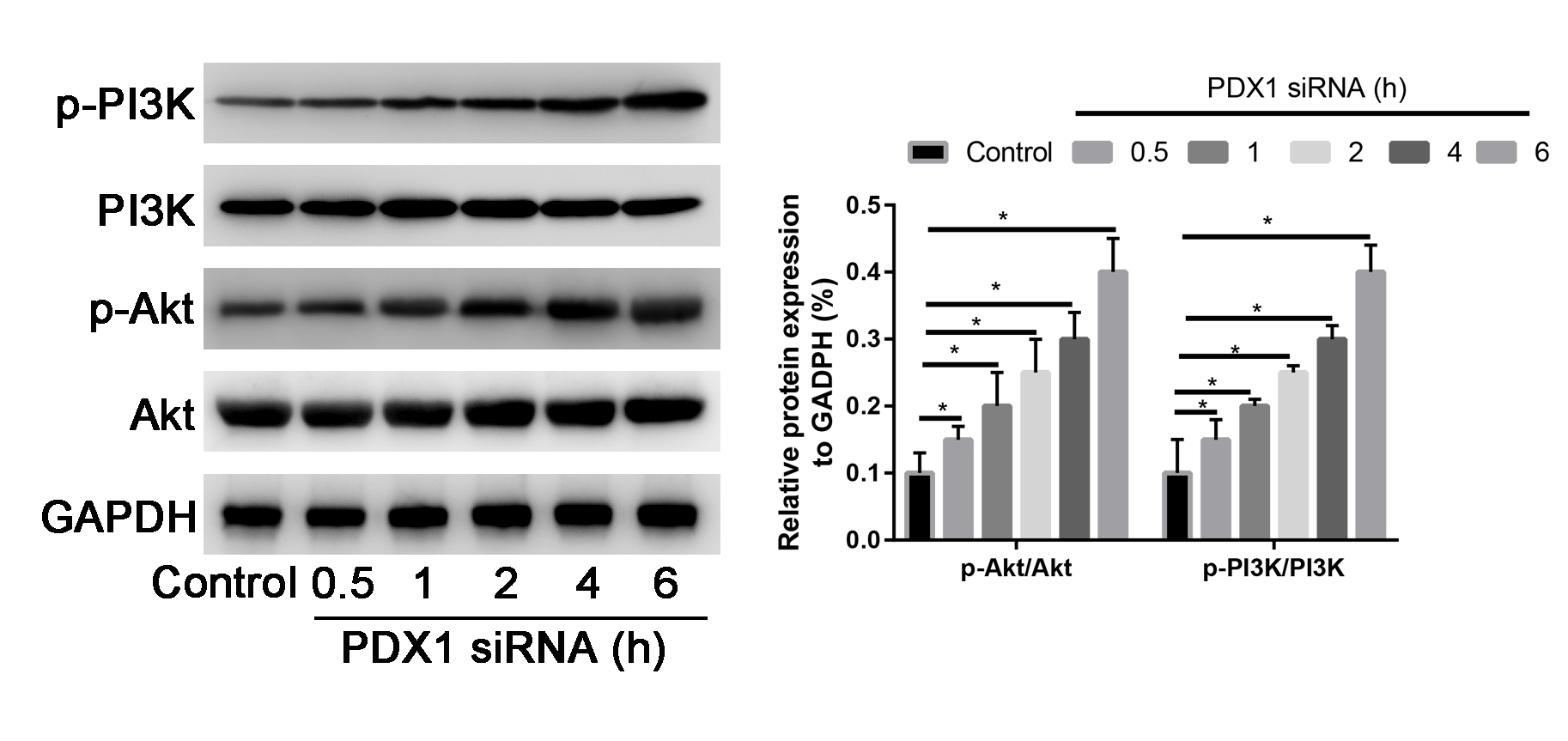

Supplement: Supplementary file 2 — Additional file 2. Relative p-PI3K, PI3K, p-Akt and Akt expression in control and PDX-1 treatment group with different treatment. [file 13018_2021_2825_MOESM2_ESM.tif]

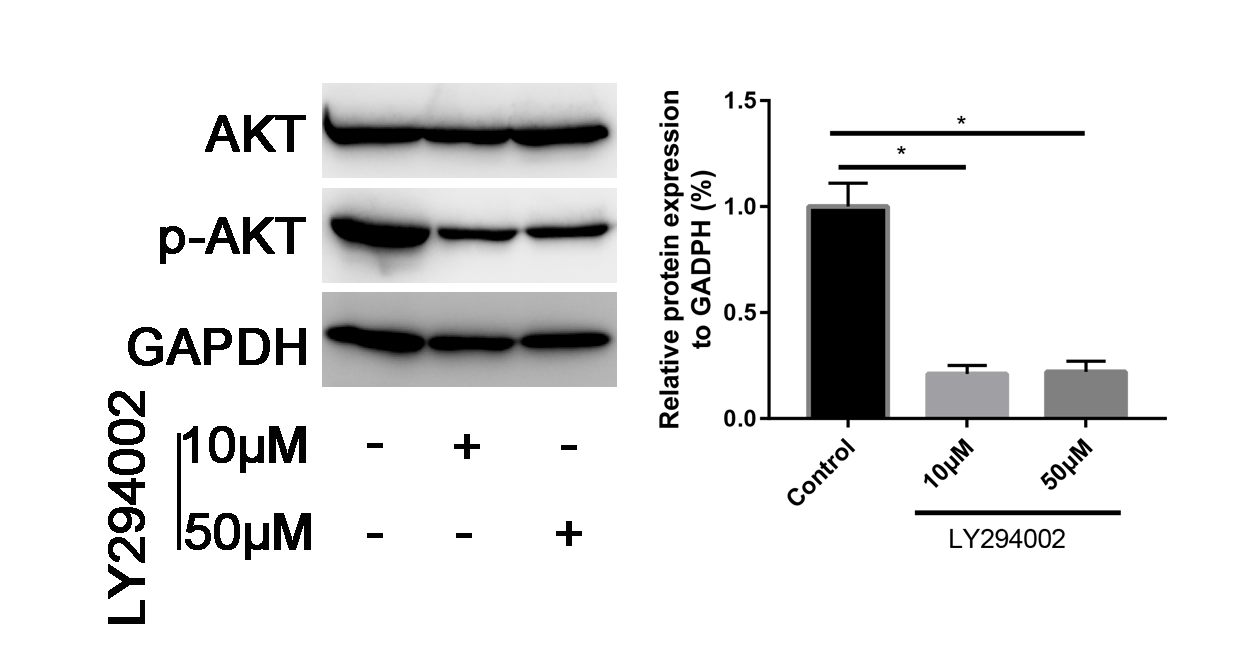

Supplement: Supplementary file 3 — Additional file 3. Relative Akt and p-Akt expression in control and LY294002 (10μM and 50 μM) groups. [file 13018_2021_2825_MOESM3_ESM.tif]
